# A closer look at the relationships between aspects of connectedness and flourishing

**DOI:** 10.3389/fpsyg.2023.1137752

**Published:** 2023-03-30

**Authors:** Martina Rahe, Petra Jansen

**Affiliations:** ^1^Institute of Psychology, University of Koblenz, Koblenz, Germany; ^2^Faculty of Human Sciences, University of Regensburg, Regensburg, Germany

**Keywords:** flourishing, self-love, pro-socialness, nature connectedness, well-being, connectedness

## Abstract

Everyone strives for personal happiness or well-being. Flourishing is a broader concept of well-being. To better understand which factors are associated to people’s flourishing, we took a closer look at the relationships of flourishing with three aspects of connectedness: Connectedness with oneself (self-love), with others (pro-socialness), and with the surrounding nature (nature connectedness). Participants were 138 adults between 18 and 71 years (*M* = 23.21, SD = 7.90, 98 women, 40 men). Significant positive correlations were found between flourishing and self-love and between flourishing and pro-socialness. Furthermore, nature connectedness correlated positively with self-love and with pro-socialness. A regression analysis revealed that all predictors explained 57.5% of the variance of the criterion flourishing. Self-love and pro-socialness were significant predictors of flourishing while nature connectedness was not. One explanation for the large correlations between self-love and flourishing could be overlapping aspects in both questionnaires. The fact that pro-socialness is a stronger predictor than nature connectedness could be due to a more reciprocal reinforcement of pro-social behavior. If a person treats another well, s/he is more likely treated well by that person which could reflect flourishing. Such a direct reciprocal relationship does not exist with nature.

## 1. Introduction

Everyone strives for personal happiness and well-being; however, the definition of those concepts is manifold. Seligman (2002) suggested that important elements of happiness were positive emotion, engagement, and meaning in life. Later, he described elements of well-being as positive emotion, engagement, relationships, meaning, and accomplishment (Seligman, 2010). Diener (1984) defined well-being by external criteria such as virtue or holiness, the evaluation of one’s life in positive terms, and as a predominance of positive affect over negative affect. Next to happiness and well-being, the concept of flourishing exists. VanderWeele (2017) suggests looking more closely at flourishing as a broader concept of well-being. He argues that flourishing includes happiness and life satisfaction, mental and physical health, meaning and purpose, character and virtue, and close social relationships. Diener et al. (2010) define flourishing as social-psychological prosperity and include human psychological needs, e.g., the need for competence, relatedness, and self-acceptance in their model of flourishing (see also Ryan and Deci, 2000).

Other authors define flourishing as one end of the mental health continuum on which the other end is languishing (Keyes, 2002) or consider flourishing “synonymous with a high level of mental well-being” (Huppert and So, 2013, p. 838). Moreover, flourishing means the presence of both hedonic (“feeling good”) and eudaimonic (“functioning well”) well-being (Schotanus-Dijkstra et al., 2016). According to this, it is evident that flourishing is a complex concept with many aspects (Hone et al., 2014). Chen et al. (2022) demonstrated that the six domains of flourishing, emotional health, physical health, meaning and purpose, character strengths, social connectedness, and financial security are independently associated with a greater composite flourishing score.

To measure flourishing, people are, for example, asked about their success in relationships, self-esteem, purpose, and optimism (Diener et al., 2010). However, there are also more complex measurements that measure the broader components of composite flourishing, for example, the 40-item flourishing index, which consists of items of former questionnaires to each of the dimensions mentioned above (Lee et al., 2020). Many studies have investigated factors that influence flourishing (Yildirim, 2019; Goldstein et al., 2021). They have found that flourishing is associated, among some socio-demographic characteristics, with high levels of extraversion and conscientiousness and low levels of neuroticism (Schotanus-Dijkstra et al., 2016). Being male, older, more educated, and married was connected to flourishing as well as superior profiles of psychosocial functioning (Keyes, 2002). Moreover, flourishing is correlated with essential need satisfaction (competency, relatedness, and autonomy) and with the factors of the Ryff scales (Ryff and Keyes, 1995, autonomy, mastery, growth, relationships, purpose, and self-acceptance) (Diener et al., 2010). Those studies provide evidence that connectedness is a relevant factor for flourishing. Wamsler and colleagues integrated the elements of compassion, empathy, kindness, and generosity into the concept of connectedness which are all related to pro-socialness and nature connectedness (Wamsler et al., 2021). According to this, it is important to investigate which aspect of connectedness is associated with people’s flourishing. For this, it is the main goal of this study to take a closer look at the relationships of flourishing with three aspects of connectedness: Connectedness with oneself (self-love), with others (pro-socialness), and with the surrounding nature (nature connectedness).

### 1.1. Self-love

Self-love is often falsely understood as narcissism (Brown and Bosson, 2001), selfishness (Fromm, 1939) or labeled as self-love but measured with a questionnaire about self-compassion or self-esteem. For example, self-compassion integrates the components of self-kindness, common humanity, and mindfulness (Neff, 2003). It is related to compassion, which develops from love when an individual suffers. Self-love develops from love without suffering. In a qualitative interview study with Spanish-speaking adults from the US, self-love was associated with well-being (Hernandez et al., 2016). Henschke and Sedlmeier (2021) define self-love as an attitude of self-kindness, including self-contact, self-acceptance, and self-care. Self-contact is defined as giving attention to and awareness of oneself whereas self-acceptance is being at peace with oneself and self-care being protective of and caring for oneself (Henschke and Sedlmeier, 2021). Regarding the relationship between self-love and the other aspects of connectedness investigated here, pro-socialness and nature connectedness, one study found positive associations between self-care and altruistic and pro-ecological behavior (Corral-Verdugo et al., 2021). The constructs in this study differ from the constructs in the present study: We used the broader concept of self-love whereas Corral-Verdugo et al. (2021) used self-care which is one component of self-love (Henschke and Sedlmeier, 2021). Moreover, altruistic behavior is only one aspect of prosocial behavior and in our study, nature connectedness was used instead of pro-ecological behavior. Nevertheless, positive relationships between the investigated variables of Corral-Verdugo et al. (2021) provide an indication that the three aspects of connectedness should be related as well. In another study using a structural model, it was shown that the factor sustainable behavior contains the constructs of self-care, altruism, and pro-ecological behavior, and sustainable behavior is related to human well-being (Torres-Soto et al., 2022).

### 1.2. Pro-socialness and nature connectedness

Prosocial behavior describes the behavior that results in the benefit for others (Eisenberg, 1982). Martela and Ryan (2016) found in an experimental study where half of the participants were told that their participation in a computer game had a prosocial effect that this induction leads to different aspects of their own well-being (vitality, meaningfulness, positive affect). Altruism, as one specific form of pro-socialness, is also associated with nature connectedness (Otto et al., 2021) and life satisfaction (Becchetti et al., 2017). Nature connectedness is associated with different aspects of well-being (vitality, autonomy, positive affect, personal growth), pro-socialness (altruistic concerns), and self-love (self-acceptance) (Nisbet and Zelenski, 2013). Moreover, a meta-analysis showed significant but small relationships between nature connectedness and eudaimonic and hedonic well-being (Pritchard et al., 2020). There was no difference between the effect sizes of the relationship between eudaimonic and hedonic well-being and nature connectedness. Especially, people who are connected to nature have higher levels of self-reported personal growth, which gives a hint for the relationship between nature connectedness and self-love. Another recent meta-analysis investigated the relationship between connectedness to nature and well-being (Wu and Jones, 2022). In that meta-analysis, sub-categories of well-being were emotional, psychological, and social well-being and flourishing was considered psychological well-being. Results showed a significantly large relationship between psychological well-being and nature connectedness (Wu and Jones, 2022). Other studies showed positive correlations between social connectedness and flourishing (Eraslan-Capan, 2016) and between nature-related schemas and flourishing (Diržytė and Perminas, 2020). Nelson et al. (2016) investigated the positive effects of a 6-week intervention of prosocial (other kindness) or self-oriented (self-kindness) behavior on flourishing and positive and negative emotions in US adults. Results showed that pro-social behavior improves flourishing over and above self-oriented behavior. Comparable results for positive and negative emotions could illustrate that pro-social behavior had stronger effects than self-oriented kindness. The present study aims to investigate the influence of three aspects of connectedness, to oneself-to others, and to nature, on peoples’ flourishing. This could help to decide which aspect of connectedness should be strengthened to enhance someone’s psychological well-being. An intervention could then be implemented to investigate its effect on adults’ flourishing.

### 1.3. The goal of the study

The study’s main goal is to investigate relationships between the three aspects of connectedness and flourishing. We choose flourishing over well-being or life satisfaction because flourishing is considered a broader concept (VanderWeele, 2017). The following hypotheses will be investigated in detail: 1. The three aspects of connectedness, self-love, pro-socialness, and nature connectedness are correlated to each other (Corral-Verdugo et al., 2021) and to flourishing (Eraslan-Capan, 2016; Nelson et al., 2016; Diržytė and Perminas, 2020; Torres-Soto et al., 2022; Wu and Jones, 2022), 2. The three aspects of connectedness predict flourishing (Eraslan-Capan, 2016; Nelson et al., 2016; Diržytė and Perminas, 2020; Torres-Soto et al., 2022; Wu and Jones, 2022), and 3. The relationship between self-love and flourishing is mediated through pro-socialness and nature connectedness.

## 2. Methods

### 2.1. Participants

Participants were 138 adults between 18 and 71 years (*M* = 23.21, SD = 7.90, 98 women, 40 men). Two participants had completed apprenticeships, 131 had a high school leaving diploma, and 5 persons had other education degrees. To determine the sample size, G*Power (Faul et al., 2007) analyses were conducted *a priori*: For the first hypothesis, medium effect sizes between the three aspects of connectedness and flourishing are assumed. Due to the multiple testing of six correlations, *p* was set to 0.0083 (Bonferroni corrected). The G*Power analysis resulted in 126 participants (1 – β = 0.80). For the second hypothesis, a medium effect size of *f*^2^ = 0.15 is assumed for the multiple regression analysis (1 – β = 0.80, α = 0.05). Therefore, 77 participants were required for the second hypothesis.

### 2.2. Material

#### 2.2.1. Flourishing

To measure flourishing, the Flourishing Scale (FS; Diener et al., 2010, German version: Esch et al., 2013) was used. The questionnaire consisted of eight items (example item: I lead a purposeful and meaningful life). Participants rated each item on a 7-point Likert Scale, ranging from 1 = *strongly disagree* to 7 = *strongly agree*. Esch et al. (2013) supported the reliability and validity of the German scale, with Cronbach’s alphas ranging between 0.79 and 0.85. In our sample, internal consistency was good (Cronbach’s alpha = 0.84, McDonald’s omega = 0.85).

#### 2.2.2. Self-love

Self-love was measured with the Self-love questionnaire (Henschke and Sedlmeier, n.d.). It consisted of 27 items (example item: I feel fine the way I am) and must be answered on a 5-point Likert scale ranging from 1 = *not true at all* to 5 = *completely true*. In a recent study, internal consistency was excellent (Cronbach’s alpha = 0.92) (Jansen et al., 2022). Internal consistency was also excellent in our sample (Cronbach’s alpha = 0.92, McDonald’s omega = 0.92).

#### 2.2.3. Pro-socialness

Prosocial behavior was investigated with the Prosocialness Scale for Adults (Caprara et al., 2005). The questionnaire contains 16 items that must be answered on a 5-point Likert scale ranging from 1 = *never/almost never true* to 5 = *almost always/always true* (example item: I try to console those who are sad.). The questionnaire is based on item response theory (IRT). Reliability (*α* = 0.91), difficulty parameter, and discrimination parameter were good, and the results of IRT analyses support effectiveness and sensitivity (Caprara et al., 2005). For the German version, the questionnaire was forward and backward translated. Internal consistency was good (Cronbach’s alpha = 0.87, McDonald’s omega = 0.87).

#### 2.2.4. Nature connectedness

Nature connectedness was measured with the Connectedness to Nature Scale (CNS, Pasca et al., 2017). It consists of 13 items, which are answered on a 5-point Likert scale ranging from 1 = *strongly disagree* to 5 = *strongly agree* (example item: Like a tree can be part of a forest, I feel embedded within the broader natural world.). In this study, three items had to be excluded because of low corrected item-total correlations (< 0.3). For the remaining 10 items, internal consistency was good (Cronbach’s alpha = 0.87, McDonald’s omega = 0.87).

### 2.3. Procedure

The survey was conducted using SoSciSurvey (Leiner, 2019). Participants received an email with the link to the study. First, all participants gave their informed consent. Then, they fill out a questionnaire regarding socio-demographics (sex, age, education), self-love, pro-socialness, nature connectedness, and flourishing. They were then thanked for their participation. The study was preregistered at osf,^1^ conducted according to the ethical guidelines of the Helsinki declaration, and approved by the Ethic Research Board of the University (no. 22-3,059-101).

### 2.4. Statistical analyses

First, correlations between the means of the three aspects of connectedness, self-love, pro-socialness, and nature connectedness, and flourishing are calculated. A multiple regression analysis (enter method) with the criterion flourishing and the predictors self-love, pro-socialness, and nature connectedness has been calculated for the second hypothesis. For the third hypothesis, we ran a mediation analysis using Process macro (Model 4, Hayes, 2018) with the criterion flourishing, the predictor self-love, and nature connectedness and pro-socialness as possible mediators. The analysis uses ordinary least squares regression, yielding unstandardized path coefficients for total, direct, and indirect effects. Bootstrapping with 5,000 samples together with heteroscedasticity consistent standard errors (Davidson and MacKinnon, 1993) were employed. Effects were regarded as significant when zero was not included in the confidence interval. For the analysis of all data, SPSS 29 was used.

## 3. Results

Correlations between all study variables are shown in Table 1. Significant positive correlations were found between flourishing and self-love, *r* = 0.734, and between flourishing and pro-socialness, *r* = 0.227. Furthermore, nature connectedness correlated positively with self-love, *r* = 0.279, and with pro-socialness, *r* = 0.352. Contrary to hypothesis 1, no significant correlations were found between flourishing and nature connectedness and between self-love and pro-socialness.

**Table 1:** Correlations between flourishing, self-love, pro-socialness, and nature connectedness.

	Flourishing	Self-love	Pro-socialness
Self-love	*r* = 0.734		
	*p* < 0.001		
Pro-socialness	*r* = 0.227	*r* = 0.063	
	*p* = 0.007	*p* = 0.461	
Nature connectedness	*r* = 0.205	*r* = 0.279	*r* = 0.352
	*p* = 0.016	*p* < 0.001	*p* < 0.001

For hypothesis 2, a regression analysis with enter method was calculated with the criterion flourishing and the predictors self-love, pro-socialness, and nature connectedness. Overall, the analysis revealed that the model was significant and all predictors explained 57.5% of the variance of the criterion, *F*(3, 134) = 60.53, *p* < 0.001. Self-love, *β* = 0.742, *p* < 0.001, and pro-socialness, *β* = 0.206, *p* < 0.001, were significant predictors of flourishing while nature connectedness, *β* = −0.075, *p* = 0.232, was not (Table 2).

**Table 2:** Regression analysis: predictors of flourishing.

	*ß*	*t*	*p*
Constant		1.321	0.189
Self-love	0.742	12.640	<0.001
Pro-Socialness	0.206	3.427	<0.001
Nature connectedness	−0.075	−1.201	0.232

Hypothesis 3 was analyzed using Process (Model 4, Hayes, 2018). Indirect effects with nature connectedness or pro-socialness as mediators of the relationship between self-love and flourishing were not significant (see Supplementary Figure S1). The total, *c* = 0.734, 95%CI [0.859, 1.179], and the direct, *c*’ = 0.742, 95%CI [0.869, 1.191], effects of self-love on flourishing, were significant. Overall, the model was significant and all variables explained 57.5% of the variance of flourishing, *R* = 0.759, *F*(3, 134) = 60.534, *p* < 0.001.

## 4. Discussion

Results of the present study showed significant associations between flourishing and two aspects of connectedness, namely self-love and pro-socialness. In line with hypothesis 1, we found strong correlations between self-love and flourishing as well as small correlations between pro-socialness and flourishing. Contrary to our assumptions, the third aspect of connectedness, nature connectedness, was not significantly correlated to self-love (after Bonferroni correction). Regarding correlations between the aspects of connectedness, nature connectedness was significantly correlated with self-love and pro-socialness, but self-love was not associated with pro-socialness. Hence, hypothesis 1 was only partly confirmed by the results. Regarding hypothesis 2, self-love and pro-socialness significantly predicted flourishing whereas nature connectedness was no significant predictor.

The correlations between self-love and flourishing are in line with results showing associations between self-love and well-being (Hernandez et al., 2016). Because the correlations in the present study were rather strong, it could be assumed that the questionnaires on self-love (Henschke and Sedlmeier, n.d.) and flourishing (Diener et al., 2010) have overlapping aspects. Especially, two items of the flourishing scale are “I am engaged and interested in my daily activities” and “I am competent and capable in the activities that are important to me” which are related to the two items of the self-love questionnaire: “I consciously decide with whom I spend my free time,” “I know which people and activities are good for me,” and “I do physical activities that are good for me.” Moreover, there are items in the self-love questionnaire (“I notice the signals of my body,” “I take time for myself”) that are prerequisites for the statements in the flourishing scale (“I am a good person and live a good live”). Apart from that there are statements in the flourishing scale regarding people’s social life (“My social relationships are supportive and rewarding,” “People respect me,” “I actively contribute to the happiness and well-being of others”) and their future (“I am optimistic about my future”) that have no corresponding statements in the self-love questionnaire. In conclusion, the strong correlation between self-love and flourishing could result from overlapping parts of the questionnaires and because aspects of self-love seem to be important to social-psychological prosperity or flourishing (Diener et al., 2010).

Pro-socialness was a positive predictor of flourishing. That result is in line with previous studies showing that prosocial behavior is connected to vitality or positive affect (Martela and Ryan, 2016) and that altruism is also associated with connectedness to life satisfaction (Becchetti et al., 2017). Moreover, the autonomous motive to help other people is associated with subjective well-being (Weinstein and Ryan, 2010).

Contrary to our hypothesis, nature connectedness was not significantly correlated to flourishing. Other studies and meta-analyses found small significant correlations between connectedness to nature and different aspects of well-being that ranged between 0.17 < *r* < 0.30 (Nisbet and Zelenski, 2013; Pritchard et al., 2020). In the present study, the correlation of nature connectedness and flourishing was within this range, too, but failed to reach significance. Another explanation for only small non-significant correlations could be that people with a higher nature connectedness worry more about the threat to the environment and their own future. Nisbet and Zelenski (2013) showed that nature connectedness was strongly associated with biospheric environmental concerns. More worry or concern could then be related to less flourishing. In the regression analysis, nature connectedness was no significant predictor of flourishing. That result could be because nature connectedness had small to medium relationships to self-love (Nisbet and Zelenski, 2013) and pro-socialness (Pirchio et al., 2021) and these factors were stronger predictors of flourishing than nature connectedness. Hence, besides self-love and pro-socialness, nature connectedness cannot explain the variance of flourishing incrementally.

Also contrary to our hypothesis, self-love was not associated with pro-socialness. Because self-love is a rather new empirically investigated concept, other studies examining self-love and prosocial behavior are rare. One study (Corral-Verdugo et al., 2021) found positive associations between altruistic behavior and self-care. Besides differences in the constructs of altruistic behavior vs. pro-socialness and self-love vs. self-care, Corral-Verdugo et al’s (2021) sample were Mexican adults from low, middle, and high socioeconomic strata. Our sample was approx. 15 years younger, from Germany, and highly educated (95% had at least 13 years of schooling).

The results of our study contribute to the existing literature on the associations between connectedness and flourishing. Self-love as a rather new construct was a strong predictor of people’s flourishing. This could be considered in interventions to enhance flourishing in healthy adults. Moreover, the correlation between pro-socialness and flourishing clarifies that it could be beneficial to encourage people to help others and engage themselves in prosocial activities. The small non-significant correlations between nature connectedness and flourishing should be analyzed in more detail in intervention studies. When higher concerns and worries in people with high nature connectedness are a reason for non-significant correlations, more nature contact could enhance people’s flourishing independent of their concerns. A mindfulness intervention could even reduce these worries (Delgado et al., 2010) to enhance the effect of a nature contact intervention.

This biased sample is a limitation of our study. Another limitation is that the questionnaires on self-love and flourishing could have overlapping items. Future research could use another measurement of flourishing that distinguishes for example the six different concepts of flourishing (Lee et al., 2020). Furthermore, other aspects of well-being, e.g., subjective well-being or life satisfaction, and other aspects of connectedness, e.g., connectedness to the transcendent could be examined. Besides this, the sample should be more diverse including, adolescents, older people, and less educated participants in the sample.

To conclude, self-love and pro-socialness had the strongest effect on people’s flourishing. For possible intervention programs to enhance flourishing in young adults, these two aspects of connectedness should be in the focus.

## Data availability statement

The datasets presented in this study can be found in online repositories. The names of the repository/repositories and accession number(s) can be found below: https://osf.io/v9mqg/files/osfstorage/63b591840b7198070b7c2d47.

## Ethics statement

The studies involving human participants were reviewed and approved by Ethic Research Board of the University Regensburg (no. 22-3059-101). The patients/participants provided their written informed consent to participate in this study.

## Author contributions

MR and PJ contributed to the study's conception and design, wrote the first draft of the manuscript, and commented on previous versions of the manuscript. Material preparation, data collection, and analysis were performed by MR. All authors contributed to the article and approved the submitted version.

## Conflict of interest

The authors declare that the research was conducted in the absence of any commercial or financial relationships that could be construed as a potential conflict of interest.

## Publisher’s note

All claims expressed in this article are solely those of the authors and do not necessarily represent those of their affiliated organizations, or those of the publisher, the editors and the reviewers. Any product that may be evaluated in this article, or claim that may be made by its manufacturer, is not guaranteed or endorsed by the publisher.

## Footnotes

^1^ https://osf.io/xma3h/?view_only=52062e62c49547858eab5d3542605f2d
