# Supplementary material for: A closer look at the relationships between aspects of connectedness and flourishing
Source: Front Psychol. 2023 Mar 30;14:1137752. doi: 10.3389/fpsyg.2023.1137752 (PMC10097885; doi:10.3389/fpsyg.2023.1137752)
Supplement: Supplementary file 1 [file Table_1.DOCX]

**Fig 1**

Mediation effect of nature connectedness and pro-socialness on the relation between self-love and flourishing (all effects are standardized). * *p* < .001.
